# *Staphylococcus aureus* infections following knee and hip prosthesis insertion procedures

**DOI:** 10.1186/s13756-015-0057-4

**Published:** 2015-05-15

**Authors:** Jean Marie Arduino, Keith S Kaye, Shelby D Reed, Senaka A Peter, Daniel J Sexton, Luke F Chen, N Chantelle Hardy, Steven YC Tong, Steven S Smugar, Vance G Fowler, Deverick J Anderson

**Affiliations:** 1 Merck & Co., Inc., Kenilworth, NJ USA; 2 Wayne State University, Detroit, MI USA; 3 Duke University Medical Center, Durham, NC USA; 4 Menzies School of Health Research, Darwin, Northern Territory Australia

**Keywords:** S. aureus infections, Hip prosthesis, Knee prosthesis, Epidemiology, Retrospective cohort

## Abstract

**Background:**

*Staphylococcus aureus* is the most common and most important pathogen following knee and hip arthroplasty procedures. Understanding the epidemiology of invasive *S. aureus* infections is important to quantify this serious complication.

**Methods:**

This nested retrospective cohort analysis included adult patients who had undergone insertion of knee or hip prostheses with clean or clean-contaminated wound class at 11 hospitals between 2003–2006. Invasive *S. aureus* infections, non-superficial incisional surgical site infections (SSIs) and blood stream infections (BSIs), were prospectively identified following each procedure. Prevalence rates, per 100 procedures, were estimated.

**Results:**

13,719 prosthetic knee (62%) and hip (38%) insertion procedures were performed. Of 92 invasive *S. aureus* infections identified, SSIs were more common (80%) than SSI and BSI (10%) or BSI alone (10%). The rate of invasive *S. aureus* infection/100 procedures was 0.57 [95% CI: 0.43-0.73] for knee insertion and 0.83 [95% CI: 0.61-1.08] for hip insertion. More than half (53%) were methicillin-resistant. Median time-to-onset of infection was 34 and 26 days for knee and hip insertion, respectively. Infection was associated with higher National Healthcare Safety Network risk index (p ≤ 0.0001).

**Conclusions:**

Post-operative invasive *S. aureus* infections were rare, but difficult-to-treat methicillin-resistant infections were relatively common. Optimizing preventative efforts may greatly reduce the healthcare burden associated with *S. aureus* infections.

## Introduction

The impact of surgical site infections (SSIs) following prosthesis procedures is devastating, often leading to higher mortality, prolonged hospitalizations, frequent readmissions, and increased costs and overall healthcare burden [1,2]. According to the United States (US) National Healthcare Safety Network (NHSN), SSI rates following total knee or hip arthroplasty procedures performed from January 1, 2006 through December 31, 2008 ranged from 0.60 to 1.60 and 0.7 to 2.4 per 100 procedures, respectively, depending on patient risk level [3]. Common factors between knee and hip procedures found to be associated with increased risk for deep incisional or organ/space SSIs include younger age, revision procedures, longer procedure duration, severity of underlying illness, hospital affiliation with a medical school, bed size >200, and trauma [4].

*Staphylococcus aureus* is the most common and most important pathogen following these and other orthopedic procedures. *S. aureus* accounts for approximately half of the deep incisional or organ/space SSIs following knee or hip joint arthroplasty reported to NHSN, with methicillin-resistant *S. aureus* accounting for 19% of these infections [5]. At a single-center [6], the reported rate of SSI was 1.1 per 100 procedures following total joint arthroplasty between 2003 to 2005, with *S. aureus* accounting for more than 50% of the infections; in addition, methicillin-resistant *S. aureus* (MRSA) accounted for 21% of SSIs following total knee arthroplasty and 31% of SSIs following total hip arthroplasty.

Using data from over 96,000 orthopedic, neurosurgical, cardiothoracic, and plastic surgical procedures performed in adults, we recently showed that *S. aureus* was the causative pathogen for about 50% of all invasive post-operative infections [7]. Of these *S. aureus* infections, SSI were diagnosed more often (70%) versus bloodstream infections (BSI) alone (30%). The overall rate of invasive *S. aureus* infections was 0.47 infections per 100 procedures (95% CI, 0.43-0.52) [7]. The rate of invasive *S. aureus* infections in the orthopedic surgery subgroup (insertion of prosthetic implants [ie, insertion of knee, hip, or other prosthesis], open reduction and internal fixation of a fracture, other musculoskeletal procedures, or amputation) in the preceding study was 0.37 infections per 100 procedures [95% CI, 0.32-0.42]. These infections were associated with three variables included in the NHSN risk index score: 1) American Society of Anesthesiologists (ASA) score; 2) wound class; and 3) length of procedure and patient age. In addition, orthopedic procedures involving prosthetic implants had a higher incidence of invasive *S. aureus* infections, 0.68 infections per 100 procedures (95% CI, 0.56-0.81), than the overall orthopedic subgroup. In the current report, we describe the incidence of invasive *S. aureus* SSI and BSI in the subset of 13,791 surgical procedures involving knee or hip prosthetic devices from the preceding cohort of 96,455 major surgical procedures. We also assessed potential risk factors for *S. aureus* infection in this group.

## Methods

### Study design and population

The study design has been previously described in detail [7]. Briefly, we previously performed a multicenter, retrospective cohort study using validated, prospectively collected surgical surveillance data for SSI and microbiologic data for BSI from nine community hospitals and two tertiary hospitals in North Carolina and Virginia from 2003 to 2006. Two surgical surveillance databases were used: the Duke Infection Control Outreach Network (DICON) surgical database and the Duke University Medical Center (DUMC) surgical database. These databases included operative variables such as patient age, date of surgical procedure, type of procedure, and NHSN risk index variables (i.e., wound class, ASA score, and length of procedure). At each hospital, all SSIs were prospectively identified by trained infection-control practitioners (ICPs) using standard definitions and methods [8]. ICPs used culture results from the clinical microbiology laboratory, readmission flags, and surgeon surveys to identify patients with potential SSIs. BSI data were identified by querying microbiological databases from each participating hospital. The Duke University Health System Institutional Review Board for Clinical Investigations (DUHS IRB) was the review board overseeing the study, and served as the IRB of record for the participating hospitals.

The study population comprised patients at least 18 years of age who underwent a major surgical procedure between January 1, 2003 through December 31, 2006 (N = 81,267 patients undergoing 96,455 surgical procedures). The nested retrospective analysis was limited to prosthetic surgical procedures (knee or hip insertion) with clean or clean-contaminated wound class (N = 13,719 procedures).

### Outcome measures

The primary outcome was invasive SSI and/or BSI due to *S. aureus*. Invasive SSIs were defined as deep incisional and organ/space SSIs diagnosed within one year following surgery, consistent with Centers for Disease Control and Prevention (CDC) defintions [8]. BSIs were defined using modified CDC criteria: at least one positive blood culture within 90 days after the procedure for all pathogens except coagulase-negative staphylococci, micrococci, Propionibacteria, diphtheroids, enterococci, viridans group streptococci, and bacilli, for which at least two positive results for cultures of blood collected during a 48-hour period were required [9]. Other outcomes of interest included methicillin susceptibility of *S. aureus* isolates and time to infection.

### Statistical analysis

This analysis was restricted to surgical procedures in patients preoperatively classified as having clean or clean-contaminated wounds. Patients could have had more than one surgery during the study period, although procedures performed after documentation of a patient's first *S. aureus* infection were excluded. Infections were attributed to the most recently performed procedure. Procedures associated with a superficial incisional *S. aureus* SSI or SSI of unknown type were also excluded.

All statistical analyses were performed using SAS software, version 9.1 (SAS Institute, Cary, NC). All statistical tests were 2-tailed, and a *P-*value of less than 0.05 was considered significant. Incidence rates were calculated as the number of infections per 100 surgical procedures. Nonparametric bootstrapping was used to generate 95% confidence intervals (CIs) for the rates. The proportions of patients with infections due to MRSA were also calculated*.* Kaplan-Meier analyses were performed to compute the median time between the date of the surgical procedure and the date of onset of infection.

To identify characteristics potentially associated with the development of invasive *S. aureus* infection, bivariable comparisons were performed between patients who developed an invasive *S. aureus* infection and those who did not. *P*-values were calculated using Pearson chi-square tests for categorical variables and the Student’s t-test for continuous variables. This analysis was hypothesis-generating; no adjustment was made for multiple statistical tests, and the results should be interpreted accordingly.

## Results

### Study population

A total of 13,719 prosthetic knee or hip insertion procedures were performed during the study period; 8,446 (62%) involved the knee and 5,273 (38%) involved the hip. Compared with patients who underwent prosthetic knee insertions, patients who underwent prosthetic hip insertions were more likely to be 60 years of age or older (66% versus 60%, *p* < 0.0001); to be at community hospitals (60% versus 53%, *p <* 0.0001); to have clean-contaminated wound class (5% versus 4%, *p* = 0.0028); and to have more severe disease, ASA scores of 3 or greater (56% versus 50%, *p* < 0.0001) (Table 1).

**Table 1:** Procedure characteristics*

		**Prosthetic knee insertion**		**Prosthetic hip insertion**	
		**(N=8,446)**		**(N=5,273)**	
**NHSN risk index**	0	2,404	(28)	1,174	(22)
	1	4,316	(51)	2,977	(56)
	≥2	1,356	(16)	984	(19)
	Missing	370	( 4)	138	( 3)
**ASA score**	1: Healthy	600	( 7)	131	( 2)
	2: Mild Systemic Disease	3,622	(43)	2,152	(41)
	3: Severe Systemic Disease	3,855	(46)	2,423	(46)
	4: Incapacitating Disease	323	( 4)	526	(10)
	5: Dying	0	( 0)	1	( 0)
	6: Emergency Care	0	( 0)	0	( 0)
	Missing	46	( 1)	40	( 1)
**Wound class**	Clean	8,142	(96)	5,029	(95)
	Clean - contaminated	304	( 4)	244	( 5)
**Age, median (years)**		63		67	
**Age category**	18-29	395	( 5)	78	( 1)
**(years)**	30-39	421	( 5)	204	( 4)
	40-49	809	(10)	609	(12)
	50-59	1,776	(21)	900	(17)
	60-69	2,347	(28)	1,082	(21)
	70-79	2,059	(24)	1,271	(24)
	80+	639	( 8)	1,129	(21)
**Hospital**	Community (N=9)	4,442	(53)	3,160	(60)
**Type**	Tertiary (N=2)	4,004	(47)	2,113	(40)

*Data shown as n (%) of patients, unless otherwise indicated.

### Rates and characteristics of invasive *S. aureus* infection

*S. aureus* caused 92 (55%) of the 167 post-operative invasive infections. Among the invasive *S. aureus* infections, SSIs were more common (80%) than SSI and BSI (10%) or BSI alone (10%). Of the 85 post-operative infections in patients undergoing prosthetic knee insertion, 48 (56%) were invasive *S. aureus* infections, of which 43 were SSIs, 4 were BSIs, and 1 was a combined SSI and BSI. Of 82 total post-operative infections in patients undergoing prosthetic hip insertion, 44 (54%) were invasive *S. aureus* infections, of which 31 were SSIs, 5 were BSIs, and 8 were combined SSI and BSI (Table 2). The overall rate of invasive *S. aureus* infection/100 procedures was 0.57 [95% CI: 0.43-0.73] following prosthetic knee insertion and 0.83 [95% CI: 0.61-1.08] following prosthetic hip insertion (*p* = 0.06). The distribution of *S. aureus* infection types in prosthetic knee insertion procedures (SSI 90%, BSI 8%, and SSI + BSI 2%) varied significantly from the distribution in prosthetic hip insertion procedures (SSI 70%, BSI 11%, and SSI + BSI 18%), *p* = 0.02.

**Table 2:** **Characteristics of invasive** ***Staphylococcus aureus*** **Infection after orthopedic surgical procedures**

	**Prosthetic knee insertion**	**Prosthetic hip insertion**
	**(N=8,446)**	**(N=5,273)**
**Rate of infection**		
Proportion of procedures	48/8,446	44/5,273
Infections per 100 procedures (95% CI)	0.57 (0.43, 0.73)	0.83 (0.61, 1.08)
**Type of infection, n (%)**		
Surgical site infection (SSI)	43 (90)	31 (70)
Bloodstream infection (BSI)	4 ( 8)	5 (11)
BSI and SSI	1 ( 2)	8 (18)
**Time to onset of infection**		
Median (IQR), days	34.0 (18.5 - 65.5)	26.0 (16.0 - 40.5)
SSI, n (%) occurring within		
30 days	20 (45)	27 (69)
60 days	32 (73)	35 (90)
90 days	36 (82)	39 (100)
120 days	40 (91)	39 (100)
150 days	40 (91)	39 (100)
180 days	40 (91)	39 (100)
1 year	44 (100)	39 (100)
**MRSA infection**	21/47 (45)	27/44 (61)
Proportion (%) of *S. aureus* infections*		

*Excludes patients for whom sensitivity data were not available (n = 1).

MRSA accounted for 53% of *S. aureus* infections following all procedures, 45% following prosthetic knee insertion and 61% following prosthetic hip insertion (*p* = 0.11). The median time to onset of invasive *S. aureus* infection was 28 days for all procedures (interquartile range (IQR): 17–54 days), 34 days (IQR: 18–65 days) for prosthetic knee insertion, and 26 days (IQR: 16–40 days) for prosthetic hip insertion. Using a year surveillance period for SSIs, 18% (8/44) of invasive *S. aureus* SSIs occurred more than 90 days after prosthetic knee insertions, while all invasive *S. aureus* SSIs occurred within 90 days after prosthetic hip insertions.

Patients who developed invasive *S. aureus* infections had higher NHSN risk index scores than did uninfected surgical patients following prosthetic knee insertion (*p* < 0.0001) and prosthetic hip insertion (*p* = 0.0001) (Table 3). Patients who developed invasive *S. aureus* infections also had higher ASA scores and significantly longer surgical procedure durations compared to uninfected patients. Age was not associated with the risk of developing an invasive *S. aureus* infection.

**Table 3:** **Comparison of patients with post-operative** ***Staphylococcus aureus*** infection and uninfected patients following orthopedic surgical procedures*

	**Prosthetic Knee Insertion**		**Prosthetic Hip Insertion**	
	***S. aureus*** **infection**	**Uninfected**	***S. aureus*** **infection**	**Uninfected**
	**(N=48)**	**(N=8,340)**	**(N=44)**	**(N=5,168)**
**NHSN risk index**				
0	5 (10)	2,395 (29)	1 ( 2)	1,170 (23)
1	19 (40)	4,268 (51)	23 (52)	2,916 (56)
≥2	22 (46)	1,311 (16)	18 (41)	947 (18)
Missing	2 ( 4)	366 ( 4)	2 ( 5)	135 ( 3)
**p-value**	<0.0001		0.0001	
**ASA score**				
1: Healthy	1 ( 2)	599 ( 7)	0 ( 0)	131 ( 2)
2: Mild systemic disease	12 (25)	3,595 (43)	8 (18)	2,133 (41)
3: Severe systemic disease	28 (58)	3,790 (45)	28 (64)	2,359 (46)
4: Incapacitating disease	7 (15)	311 ( 4)	7 (16)	506 (10)
5: Dying	0 ( 0)	0 ( 0)	0 ( 0)	1 (<1)
6: Emergency care	0 ( 0)	0 ( 0)	0 ( 0)	0 ( 0)
Missing	0 ( 0)	45 (<1)	1 ( 2)	38 ( 1)
***p*** **-value**	0.0002		0.024	
**Wound class**				
Clean	47 (98)	8,042 (96)	43 (98)	4,927 (95)
Clean - contaminated	1 ( 2)	298 ( 4)	1 ( 2)	241 ( 5)
***p*** **-value**	0.58		0.45	
**Procedure duration**				
Mean (SD), minutes	158.2 (108.1)	124.7 (110.0)	169.3 (112.8)	125.5 (109.6)
***p*** **-value**	0.036		0.0082	
**Age category (years)**				
18-29	1 ( 2)	393 ( 5)	1 ( 2)	77 ( 1)
30-39	2 ( 4)	418 ( 5)	1 ( 2)	203 ( 4)
40-49	8 (17)	793 ( 9)	4 ( 9)	598 (12)
50-59	12 (25)	1,752 (21)	7 (16)	883 (17)
60-69	9 (19)	2,318 (28)	8 (18)	1,060 (20)
70-79	11 (23)	2,039 (24)	6 (14)	1,250 (24)
80+	5 (10)	627 ( 7)	17 (39)	1,097 (21)
***p*** **-value**	0.46		0.17	

*Data shown as n (%) of patients, unless otherwise indicated.

## Discussion

Approximately 1 million primary knee and hip arthroplasty procedures are performed annually in the US. In 2010, for example, 719,000 total knee replacements and 332,000 total hip replacements were performed [10]. The volume of joint arthroplasty procedures is projected to increase substantially in the United States in the coming decades [11]. An important complication of joint arthroplasty procedures is post-surgical infection [12], which is often caused by *S. aureus*.

The purpose of this analysis was to determine the epidemiology of invasive *S. aureus* infections following prosthetic knee or hip insertion procedures. We found a slightly higher rate of invasive *S. aureus* infections in patients receiving prosthetic hip insertions compared to those receiving prosthetic knee insertions (0.83 vs 0.57 per 100 procedures), although the difference was not statistically significant. NHSN risk index score, ASA score, and procedure duration all significantly affected the rates of postoperative staphylococcal infections for both types of prosthetic insertion procedures. In addition, more than half of the *S. aureus* infections were MRSA infections.

Our results are similar to previously published reports. Rao and colleagues found that the overall SSI rate after total joint arthroplasty at a single institution was 1.1 per 100 procedures, with *S. aureus* accounting for 53% of SSIs following total knee arthroplasty and 65% of SSIs following total hip arthroplasty [6]. Our results are also consistent with national surveillance rates, in which the deep incisional or organ/space SSI infection rates are reported to range between 0.60 to 2.40 per 100 procedures for prosthetic knee and hip insertions [3], with approximately 50% of these infections attributable to *S. aureus* and 40% of the *S. aureus* infections caused by MRSA [5].

The CDC updated the NHSN surveillance definitions for SSIs in January 2013. Prior to 2013, post-operative surveillance for an SSI continued for a year for prosthetic knee or hip insertion procedures [9]. In the new 2013 SSI definitions, the surveillance period for these procedures has been reduced to 90 days [13]. Changing the SSI surveillance definition from 1 year to 90 days results in an 18% decrease in the number of SSI following prosthetic knee insertion procedures in this study. The surveillance definitions used in estimating rates, whether pre- or post-January 2013, will need to be accounted for when interpreting time-trends in SSI rates [14].

Our study has several limitations. First, as with the primary analysis [7], true incidence rates of post-operative *S. aureus* infections were likely underestimated for the following reasons: superficial incisional SSIs and other invasive infections such as pneumonia were excluded; patients who died before diagnosis of invasive infection were not identified; and infections occurring post-discharge from the hospital may have been under-reported [15]. To minimize the likelihood of missed SSIs, surveillance methods for SSIs at study hospitals included flagging patients for re-admission and surgeon surveys to identify potential SSIs. Since BSIs were identified from the study hospitals’ microbiology databases, only patients with post-discharge BSIs re-admitted to the same study hospital performing the surgical procedure would be detected. In addition, we were unable to determine the source of BSI (e.g., “primary” versus “secondary”). The classification of surgery as elective or emergency was not captured in the surveillance database. We used clean and clean-contaminated wound class as a proxy for elective surgery. Further, patient-level data on antimicrobial prophylaxis prior to surgery were not available. Finally, the generalizability of these findings is limited since (1) all the participating hospitals are located in the southeastern U.S., and (2) the data were collected from 2003–2006, prior to improvements in infection control practices that have resulted in a reduction in MRSA infections nationally since 2006 [16].

Our study has several strengths. The data were from a large, multicenter study using pre-existing, prospectively collected surgical surveillance data for SSI and microbiology data for BSI. The study utilized well-defined methods for classification of SSI according to Centers for Disease Control and Prevention. Finally, we included data from both community and tertiary care hospitals, thereby increasing the generalizability of our results.

The healthcare and economic burden associated with prosthetic joint infections are substantial. Healthcare resource use and cost of care are significantly increased due to longer hospital stays, re-hospitalizations, lengthy antibiotic treatment, further surgery (e.g., debridement, exchange or resection arthroplasty, and amputation), rehabilitation, outpatient and emergency visits. Treatment costs for an infected knee or hip arthroplasty are more than 3 times higher than the costs for primary arthroplasty [17-20]. Assuming an infection rate of 1.0% and a cost of $130,000 per patient, the annual cost to the US healthcare system for the management of SSI after total knee arthroplasty has been estimated at ~ $2 billion [21]. Methicillin-resistant infections result in significantly higher costs of care compared to methicillin-susceptible infections, due to more hospital visits and longer hospital stays [22]. Infectious complications of arthroplasty also negatively impact the patients’ physical functioning and health-related quality of life, which potentially contribute to lost work productivity [2].

## Conclusions

We found that patients undergoing hip and knee prosthetic joint surgery are at relatively high risk for *S aureus* infections. The rates of these infections following joint arthroplasty surgery were similar to the rates following cardiac procedures (0.79 per 100 [95% CI: 0.61-0.97]) and neurosurgical procedures (0.62 per 100 [95% CI: 0.53-0.72]), assessed in the same study population [7]. Moreover, approximately 50% of the infections observed after joint arthroplasty were due to MRSA. Given the poor outcomes and high costs associated with these infections, this patient population represents a good target for interventions to mitigate risk for invasive postoperative *S. aureus* infections.
